# Dissecting the resilience of barley genotypes under multiple adverse environmental conditions

**DOI:** 10.1186/s12870-023-04704-y

**Published:** 2024-01-02

**Authors:** Ahmed M. Abdelghany, Sobhi F. Lamlom, Mahmoud Naser

**Affiliations:** 1 Crop Science Department, Faculty of Agriculture, Damanhour University, Damanhour, 22516 Egypt; 2 Plant Production Department, Faculty of Agriculture Saba Basha, Alexandria University, Alexandria, 21531 Egypt; 3 Ministry of Agriculture and Rural Affairs Key Laboratory of Soybean Biology (Beijing), Institute of Crop Sciences, Chinese Academy of Agricultural Sciences, Beijing, 100081 China

**Keywords:** Barley (*Hordeum vulgare* L.), Stability index (MTSI), Climate resilience, Environmental variability, Heat stress

## Abstract

As climate change increases abiotic stresses like drought and heat, evaluating barley performance under such conditions is critical for maintaining productivity. To assess how barley performs under normal conditions, drought, and heat stress, 29 different varieties were examined, considering agronomic, physiological, and disease-related characteristics. The research was conducted in five environments: two normal environments in 2020/2021 and 2021/2022, two drought stress environments in 2020/2021 and 2021/2022, and one heat stress environment in 2021/2022. The results demonstrated that genotype and environment significantly influenced all traits (p < 0.05), except canopy temperature, while genotype x environment interaction significantly influenced most traits, except total chlorophyll content and canopy temperature. Heat and drought stress environments often resulted in reduced performance for traits like plant height, spike length, grains per spike, and 100-grain weight compared to normal conditions. Based on individual traits, genotypes 07UT-44, 06WA-77, 08AB-09, and 07N6-57 exhibited the highest grain yield (4.1, 3.6, 3.6, and 3.6 t/ha, respectively). Also, these genotypes demonstrated enhanced stability in diverse drought and heat stress conditions, as assessed by the mean performance vs. stability index (Weighted Average of Absolute Scores, WAASB). The multi-trait stability index (MTSI) identified 07UT-44, 07UT-55, 07UT-71, and 08AB-09 as the most stable genotypes in terms of the performance of all traits. The imported lines demonstrated superior performance and stability, highlighting their potential as valuable genetic resources for developing climate-resilient barley.

**Supplementary Information:**

The online version contains supplementary material available at 10.1186/s12870-023-04704-y.

## Introduction

The impact of climate change and varying environmental conditions has resulted in the imposition of multiple abiotic and biotic stress factors on field crops [1–3]. The most practical method for addressing the anticipated climatic changes is the cultivation of field crops that are resilient to abiotic stress. Among the available crop varieties, barley (*Hordeum vulgare* L.) has demonstrated the potential to serve as a source of genes for abiotic stress tolerance, thereby enabling it to thrive in diverse environmental conditions [4]. Barley exhibits moderate levels of tolerance to drought stress, which is advantageous given the limited availability of irrigation water [5]. When compared to certain other regions of the world where rainfall can reach 200 mm, Egypt averages less than 130 mm of yearly precipitation [6]. Barley is vital for newly reclaimed soils in Egypt’s North Coastal Region [7]. It’s not just used as food for people and animals anymore – it’s also important for making malt, beer, and biodiesel. In 2020/2021, production was 160.53 million tons, but it fell to 147.05 million tons in 2021/2022 [7, 8]. However, the productivity of rainfed barley in Egypt is higher than the global average productivity [9]. Thus, the challenging environmental conditions prevalent in Egypt have necessitated the import of barley genotypes by breeders to enable adaptation to low-input conditions.

Furthermore, rising temperatures pose a major threat to barley production in Egypt’s hot climate. Average temperatures across the country’s main barley-growing regions are projected to increase by 1.5-2 °C by 2050 [10]. This warming trend exacerbates heat stress during the sensitive developmental stages of the barley crop. Acute heat waves also impose short episodes of severe heat stress [11], as exposure to unfavorable high temperatures during critical developmental stages, such as before flowering, can additionally stimulate sterility and yield failures [12, 13]. Heat stress and associated climate extremes are destructive forces that diminish yields through both direct and indirect means, underscoring the need for prompt action to expand resilience. Particularly, such rising temperature and intensified heat waves pose a major threat to barley cultivation in Egypt [14]. Hence, adaptive research and the development of resilient barley varieties capable of thriving under climate volatility and securing production are vital to maintaining high productivity.

In the face of climate change, the complex endeavor of simultaneously selecting barley genotypes with desired traits is compounded by the necessity to rapidly cultivate superior genotypes to address the increasing worldwide food needs. Hence, plant breeders often face the challenge of selecting the best statistical model for predicting genetic values while considering multiple traits [15]. To address this concern, breeders often depend on data from multi-environment trials during the later phases of the breeding process to make well-informed selections [16]. Recently, the weighted average absolute scores of best linear unbiased predictions (WAASB) represents a novel quantifiable genotype stability measure [17]. It utilizes the singular value decomposition of the BLUP matrix from a linear mixed model to dissect genotype-by-environment interaction influences [17]. Plotting the WAASB against the trait means facilitates the interpretation of both stability and productivity simultaneously, thereby enabling selection for broad adaptation.

Nevertheless, the incorporation of multiple traits enhances the precision of genotype selection. Hence, a new and rapid technique has been developed to analyze multi-environment trials, integrating the simultaneous selection for multi-trait stability into a single and easily understandable index [16]. The technique is called the multi-trait stability index (MTSI), which represents a theoretical framework that involves the simultaneous selection of genotypes based on their mean performance and stability across multiple environmental trials [16, 18]. A lower MTSI score indicates greater stability of genotypes, considering various variables. The efficacy of the MTSI has been substantiated in the identification of superior and resilient soybean genotypes across stress conditions, including drought and salinity [19, 20].

In this study, we assessed imported barley genotypes alongside locally adapted cultivars across various environmental conditions. The key goal of this investigation is to identify barley genotypes that perform exceptionally well in terms of their agronomic, physiological traits, and disease resistance while maintaining consistent performance across various challenging environments. Past research has explored heat or drought tolerance independently in barley genotypes from other regions. Our study, however, is the first to assess responses to both heat stress and water limitation in priority Egyptian breeding materials, comprising leading commercial cultivars and exotic barley materials. By pinpointing drought- and heat-resilient barley genotypes that maintain superior performance under varied sowing dates and restricted irrigation, our findings can facilitate future molecular or transgenic approaches to breed resilient varieties. The top heat-tolerant exotic lines identified through our analysis have the potential for crossing with local cultivars to increase yield potential while preserving genetic diversity for continual adaptation.

## Materials and methods

### Plant materials and experimental conditions

The study was carried out in a total of five environments: two normal environments during the 2020/2021 and 2021/2022 growing seasons, two drought stress environments during the 2020/2021 and 2021/2022 seasons, and one heat stress environment during the 2021/2022 season.

The trials were carried out at El-Bostan Experimental Farm (30˚48′65″ N, 30˚7582″ E), Faculty of Agriculture, Damanhour University, Egypt. The experimental plant materials comprised 29 barley genotypes, which consisted of 25 barley lines obtained from the University of Minnesota in Minnesota, USA. In addition, more detailed information about phenotypic characterization for these barley materials can be retrieved from the T3/barley website: https://barley.triticeaetoolbox.org/. In addition, there were four check cultivars (“Giza123”, “Giza127”, “Giza134”, and “Giza136”) from the Agriculture Research Center (ARC) in Egypt. The name, pedigree, breeding program, row type, and origin of the 29 barley materials used in this study are shown in Table 1.

**Table 1:** Name, pedigree, breeding program, row type, and origin for barely materials used in the current study

Name	Pedigree	Program	Row type	Origin
06BA-06	Crystal/91Ab3203	USDA-Aberdeen	2	USA
06N6-84	Crystal/Merit	USDA-Aberdeen	2	USA
06WA-77	91Ab6526/90Ab321	USDA-Aberdeen	2	USA
07AB-10	96Ab8309/Steffi	USDA-Aberdeen	2	USA
07AB-29	95Ab15156/M105	USDA-Aberdeen	6	USA
07AB-36	92Ab5697/95Ab15166	USDA-Aberdeen	6	USA
07MN-02	MN00-60 / MN00-52	University of Minnesota	6	USA
07N6-11	Drummond/ND17643	North Dakota State University	6	USA
07N6-57	ND19495/ND19651	North Dakota State University	6	USA
07UT-01	WA 8608-97/Baronesse	Washington State University	2	USA
07UT-36	98Ab12362/Creel	USDA-Aberdeen	6	USA
07UT-44	CI 361/ND15477	USDA-Aberdeen	6	USA
07UT-48	M105/93Ab375	USDA-Aberdeen	6	USA
07UT-55	98Ab12362/Drummond	USDA-Aberdeen	6	USA
07UT-71	92Ab5180/Drummond	USDA-Aberdeen	6	USA
07UT-86	98Ab12407/UT4467	USDA-Aberdeen	6	USA
07UT-96	6B97-2232 // 6B94-8253 / 6B97-2245 /3/ 6B98-9438 // 6B94-8253 / 6B97-2232	Busch Agricultural Resources Inc.	6	USA
08AB-09	MERIT/2B97-4077	Busch Agricultural Resources Inc.	2	USA
08MN-15	6B98-9558 / M99-2	Busch Agricultural Resources Inc.	6	USA
08N6-05	Bob/Merit//CDC Select	Washington State University	2	USA
08N6-94	94Ab13449/M103	USDA-Aberdeen	6	USA
08UT-19	Z005J004J / CORK // B1215 / Z078H050i	Busch Agricultural Resources Inc.	2	USA
08WA-40	6B00-0906/6B98-9558	Busch Agricultural Resources Inc.	6	USA
09AB-94	Kendall/Harrington	Montana State University	2	USA
09MT-02	ND19655/ND20477	North Dakota State University	6	USA
Giza 123	GIZA117/FAO86	ARC, Egypt	6R	Egypt
Giza 127	W12291/Bags//Harmal-02	ARC, Egypt	2R	Egypt
Giza 134	Alanda-01/4/WI 2291/3/Api/CM67//L2966-69	ARC, Egypt	6R	Egypt
Giza 136	PLAISANT/7/CLN-B/LIGEE640/3/S.P-B//GLORIAAR/ COME B/5/ FALCONBAR/6/LINOCLN-B/A/S.P-/LIGNEE640/3/S.P-B//GLORIABAR/COME B/5/FALCONBAR/6/LINO	ARC, Egypt	6R	Egypt

For the two normal environments (N_2021 and N_2022), full irrigation scenario was applied, and the sowing date was November 15th. Regarding the two drought stress environments during the 2020/2021 (DS_2021) and 2021/2022 (DS_2022), irrigation was withheld after the heading stage, where the sowing date was also on November 15th. To impose heat stress on the barely materials during the 2021/2022 season (HS_2022), all barley genotypes were sown on January 1st to ensure exposure to rising spring temperatures during later vegetative phases, exposing post-anthesis and grain-filling stages to excess warmth. For each environment, the experimental design used was a randomized complete block design, with each environment having three replicates. The experimental unit for the study was a plot size of 3.75 m^2^ consisting of four rows, 1.5 m wide and 2.5 m long. The seeding rate used was 119 kg per hectare. Soil samples were collected from a depth of 30 cm and analyzed according to Black et al. [21]. The physical and chemical properties of soil samples of El-Bostan experimental site in the 2020/2021 and 2021/2022 growing seasons are presented in Table S1. The meteorological data used in the study are also provided in Table S2.

### Studied traits

The study involved the measurement of nine characteristics, including the number of days to flowering (NDF), which was determined as the time taken for 50% of the spikes in a plot to extrude anthers, noted as days from January 1st. Plant height (PH, cm) was determined by measuring the length from the soil surface to the tip of the spike at harvest time using a random sample of ten plants in each plot. To measure spike length (SL), ten spikes were randomly selected at harvesting from the middle rows of plants. The length of each spike was measured in cm using a graduated ruler, and then the lengths were summed and divided by ten to obtain the average spike length. The mean number of grains per spike (NGS) for each plot was determined by randomly selecting 10 spikes from each plot. To calculate the 100-grain weight (HGW, g), 100 seeds were randomly collected from each genotype and weighed. Grain yield (GY, t/ha) was calculated by harvesting and weighing the four rows of each plot, then expressing it as tons per hectare (t/ha).

The total chlorophyll content (TCC) of the leaves was evaluated by utilizing a spad-502 chlorophyll meter (Minolta, Japan). To measure the canopy temperature (CT, °C), a portable infrared thermometer (KM 843, Comark Ltd., Hertfordshire, UK) with a field view of 100 to 1000 mm was utilized. Data on canopy temperatures were collected from the same side of each plot at 1 m from the edge and roughly 50 cm above the canopy at a 30° angle to the horizontal. On bright days, readings were taken between 1300 and 1500 h. For disease assessment, the evaluation of leaf rust (LR) disease was carried out after the flowering stage through visual scoring, where the percentage of infected leaf area was determined for every barley plot grown under open field conditions, as previously documented by Naser et al. [22].

### Statistical analysis

Data were statistically analyzed using the analysis of variance procedures by SAS 9.2 [23] for a randomized complete block design as follows:

$$\bf \text{Y}\text{i}\text{j} = {\mu } + \text{B}\text{j} + \text{G}\text{i} + \text{e}\text{i}\text{j}$$

where *Y*_*ij*_ is the measured traits; *µ* is the mean of population; *G*_*i*_ is the effect of genotype *i*; *B*_*j*_ is the effect of block *j*; and *e*_*ij*_ is the experimental error.

The linear model for an across-environments combined analysis of variance was conducted as follows:

$$\bf \text{Y}\text{i}\text{j}\text{k} = {\mu } + \text{E}\text{i} + \text{B}\text{k}\left(\text{E}\text{i}\right) + \text{G}\text{j} + \left(\text{G}\text{E}\right)\text{i}\text{j} + \text{e}\text{i}\text{j}\text{k}$$

where *µ* is a population mean; *Ei* is the effect of environment *i*; *Gj* is the effect of genotype *j*; (*GE*)*ij* is the interaction effect of *j* genotype x *i* environment; *B*_*k*_ is the effect of block k in environmental i; and e*ijk* is the experimental error.

The genotypic stability of each genotype was quantified by the WAASB from the singular value decomposition of the matrix of best linear unbiased predictions for the GEI effects generated by a linear mixed-effect model [16], estimated as indicated in Eq. (2):

$${\mathbf{W}\mathbf{A}\mathbf{A}\mathbf{S}\mathbf{B}}_{\mathbf{i}}={\sum }_{\mathbf{k}=1 }^{\mathbf{p}}\left|{\mathbf{I}\mathbf{P}\mathbf{C}\mathbf{A}}_{\mathbf{i}\mathbf{k} }{\mathbf{E}\mathbf{P}}_{\mathbf{k}}\right|\diagup{\sum }_{\mathbf{k}=1 }^{\mathbf{p}}{\mathbf{E}\mathbf{P}}_{\mathbf{k}}$$

where WAASB_i_ is the weighted average of absolute scores of the ith genotype; IPCA_ik_ is the score of the ith genotype in the *k*th interaction principal component axis (IPCA); and EP_k_ is the amount of the variance explained by the kth IPCA. The genotype with the lowest WAASB value is considered the most stable, showing the least deviation from the average performance across environments [16].

To estimate the multi-trait stability index (MTSI) [16], Eq. (3) below was used as follows;

$$\bf \bf \bf {\mathbf{M}\mathbf{T}\mathbf{S}\mathbf{I}}_{\mathbf{i}}={\left[{\sum }_{\mathbf{j}=1}^{\mathbf{f}}{\left({\mathbf{F}}_{\mathbf{i}\mathbf{j}}-{\mathbf{F}}_{\mathbf{j}}\right)}^{2}\right]}^{0.5}$$

where MTSI is the multi-trait stability index for the i_th_ genotype, F_ij_ is the jth score of the i_th_ genotype, and F_j_ is the *j*th score of ideotype. The genotype with the lowest MTSI is, therefore, closer to the ideotype and hence has a high mean performance and stability for all variables studied.

The homogeneity of variance among various environments was determined using Bartlett’s test, following the methodology outlined by Steel and Torrie [24]. Subsequently, combined analyses of variance were conducted for environments with consistent variances [25]. To compare means, we employed the HSD Tukey test with a significance level set at p ≤ 0.05. We conducted a cluster analysis of the genotypes across all traits and five environments, utilizing Ward’s method based on Euclidean distance [26]. Additionally, we calculated correlation coefficients using the Pearson correlation coefficient. Boxplots were performed with *ggplot2* package, while WAASB, MTSI, and Pearson correlation analyses were conducted using the *metan* package, whereas the *factoextra* package was manipulated to visualize the cluster analysis via R software (version 4.1.0, R Core Team in 2021).

## Results

### Analysis of variance

The combined analysis of variance (Table 2) showed that genotype (GEN) and environment (ENV) had a highly significant (p < 0.001) effect on all the studied traits, whereas the effect of genotype was significant on all the studied traits except for CT. For the effect of genotype x environment interaction, all the studied traits were significantly affected, while TCC and CT showed non-significant effects by such interaction.

**Table 2:** Combined analysis of variance for 9 studied traits of 29 barely genotypes across five environments

Source of Variance	DF	NDF	PH	TCC	CT	LR	SL	HGW	NGS	GY
ENV	4	22,800***	70,200***	2740***	2780***	12,100***	125***	25***	5040***	38.9***
REP(ENV)	5	15.4 ns	37.1NS	161*	23.6**	588***	2.25	0.228ns	92.9 ns	0.232
GEN	28	90.6 ***	422***	129***	7.71ns	774***	5.44***	1.3***	1520***	2.48***
GEN:ENV	112	23.2**	85.9***	41.1 ns	7.7ns	373***	1.53*	0.36***	198***	0.802*
Residuals	140	14.3	46.9	52.8	6.41	87.1	1.04	0.189	42	0.575
CV (%)		4.5	6.63	17.9	13.2	19.1	16.9	9.96	12.7	24.9
Mean		83.9	103.28	40.49	19.2	4.88	6.05	4.36	51.01	3.04

NDF, number of days to flowering; PH, plant height; NGS, number of grains/spike; HGW, 100-grain weight; LR, leaf rust; GY, grain yield; CT, Canopy temperatures; TTC, total chlorophyll content; LR, leaf rust. *, **, and ***: Significant at p < 0.05, < 0.01 and < 0.001

### Variation among five different environments in examined traits

**Fig. 1:**
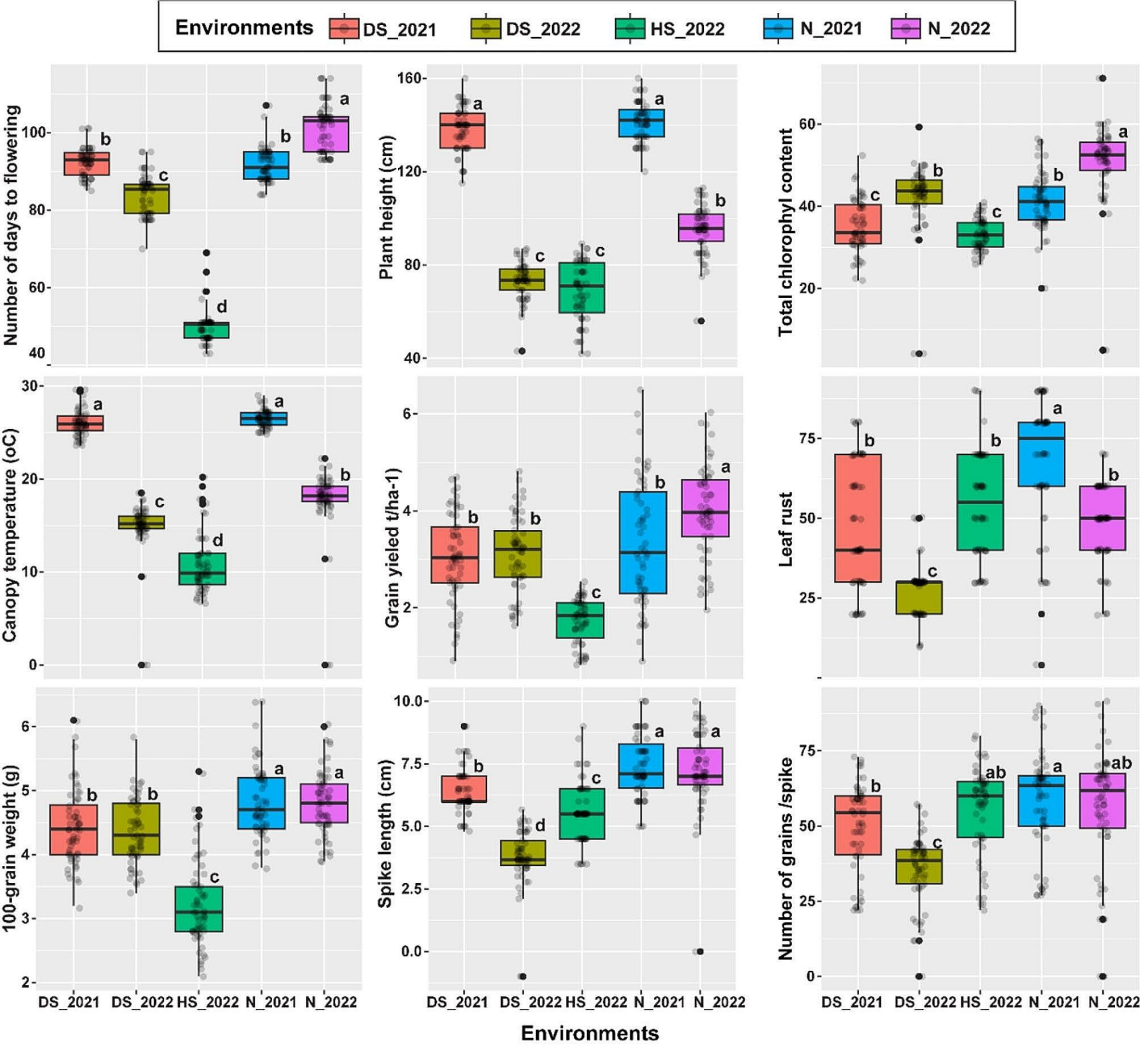
Comparisons of nine agronomic, physiological, and pathological traits among five environments. different lowercase letters indicate statistically significant differences as obtained by Tukey HSD test; p < 0.05). N_2021 and 2022 N_2022: normal conditions in 2021; DS_2021 and 2022 DS_2022: drought stress conditions in 2021; HS_2022: heat stress conditions

The average performance of nine traits across five distinct environments is presented in Fig. 1. Notably, there were significant differences observed among these five environments for each of the nine traits under investigation. For NDF, the environment that performed the best was N_2022, with 101.31 days. In contrast, the HS_2022 environment exhibited the lowest NDF value at 50.22 days. Concerning PH, the tallest barley plants (141.24 cm) were observed in the N_2021 environment, while the shortest plants (68.66 cm) were found in the HS_2022 environment. When examining physiological and pathological attributes, the highest CT (26.48 ◦C) was recorded in the N_2021 environment, while the highest TCC (50.59) was observed in the N_2022 environment. Conversely, the lowest CT (10.8 ◦C) and TCC (33.13) values were recorded in the HS_2022 environment. In terms of LR susceptibility, the lowest level (27.57) was exhibited by the DS_2022 environment, whereas the N_2021 environment displayed the highest susceptibility to LR (67.66). Regarding yield and its components, the N_2021 environment demonstrated the highest averages for SL (7.421 cm), HGW (4.83 g), and NGS (58.05). Conversely, the DS_2022 environment had the lowest values for both SL (3.76 cm) and NGS (35.34), while the HS_2022 environment recorded the lowest HGW (3.22 g). For grain yield, there was a significant difference among the five environments. The N_2022 environment produced the highest grain yield, measuring 3.98 t/ha, whereas the HS_2022 environment yielded the lowest grain yield, with an average of 1.73 t/ha.

### Variation among barley genotypes and traits across individual environments

**Fig. 2:**
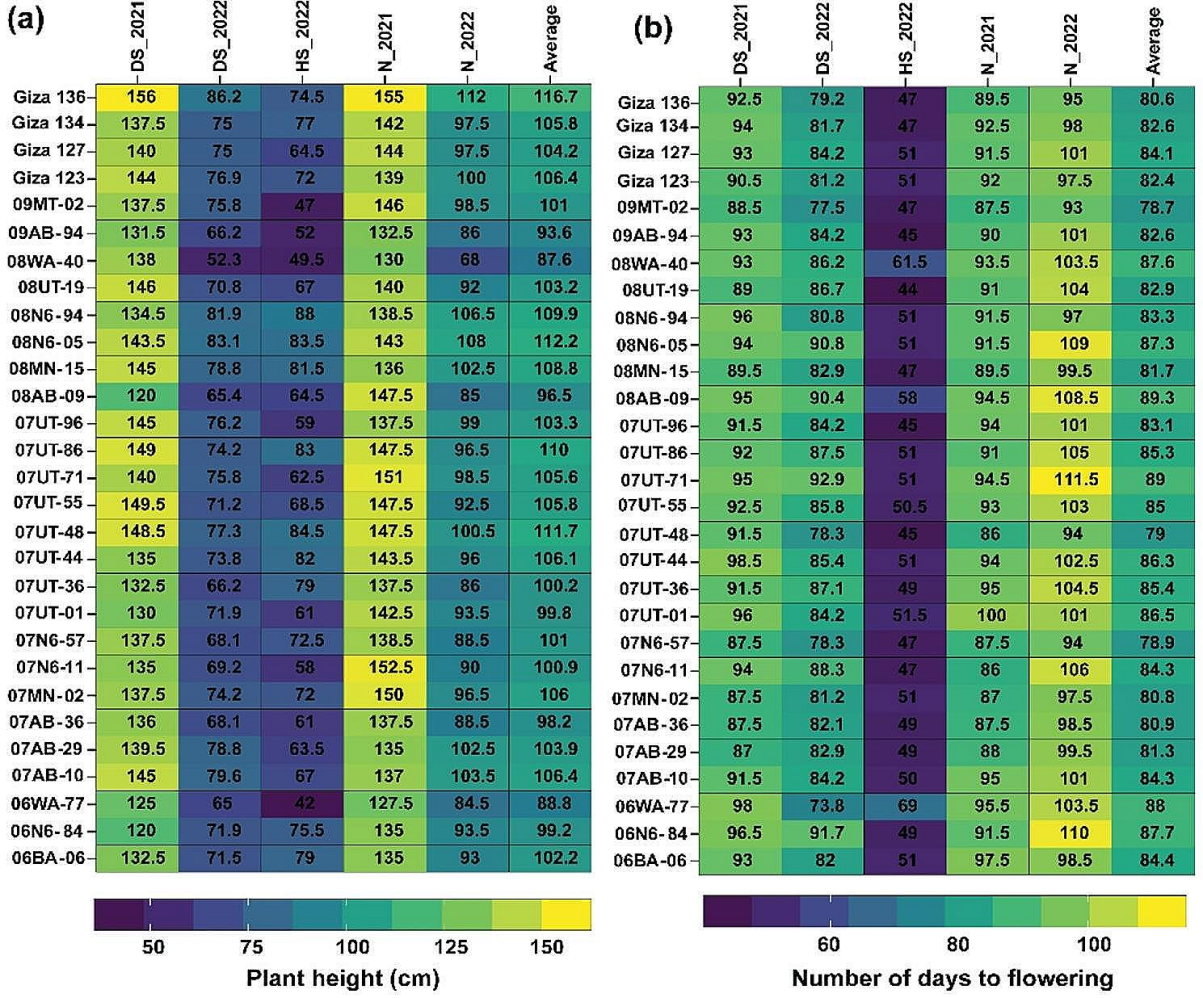
Performance of 29 barley genotypes observed in five environments for plant height (**a**) and number of days to flowering (**b**). The data represented in each column indicates the average values of each trait of barely genotypes in each of the five environments: normal conditions in 2021 (N_2021) and 2022 (N_2022), drought stress conditions in 2021 (DS_2021) and 2022 (DS_2022) and heat stress conditions (HS_2022).

**Fig. 3:**
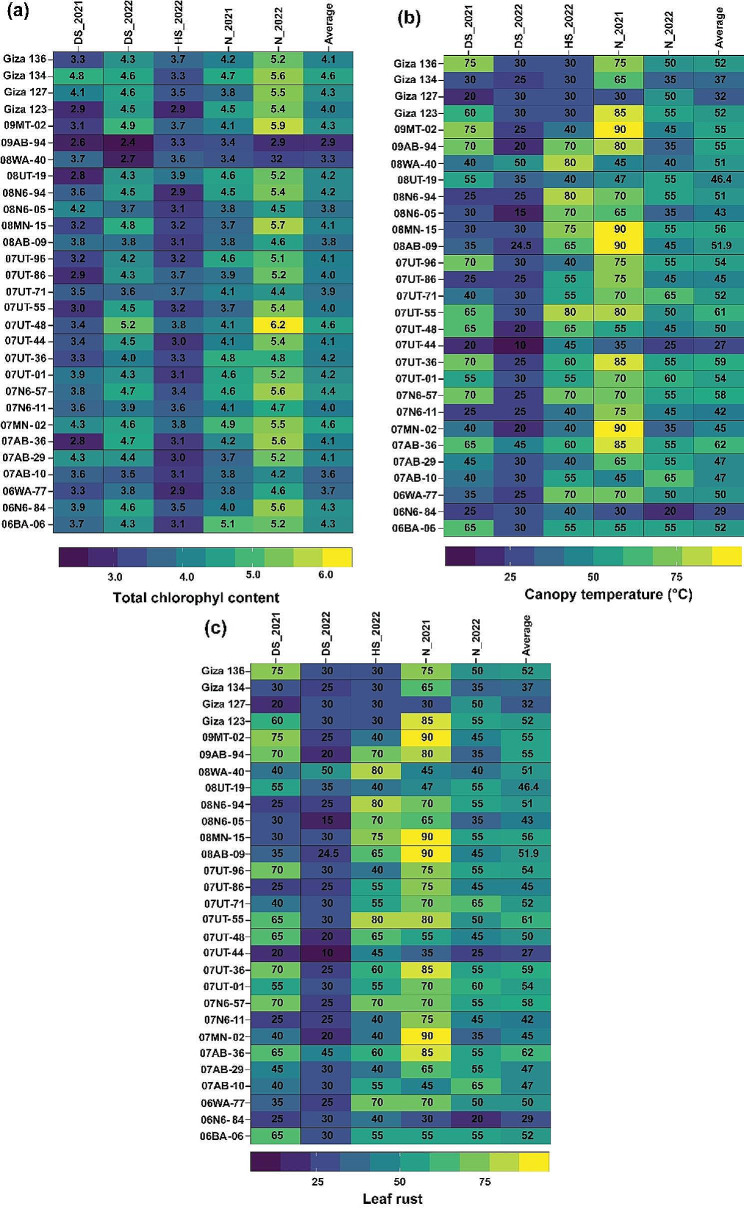
Performance of 29 barley genotypes observed in the five environments for total chlorophyl content (**a**), canopy temperature (**b**), and leaf rust (**c**). The data represented in each column indicates the average values of each trait of barely genotypes in each of the five environments: normal conditions in 2021 (N_2021) and 2022 (N_2022), drought stress conditions in 2021 (DS_2021) and 2022 (DS_2022) and heat stress conditions (HS_2022)

**Fig. 4:**
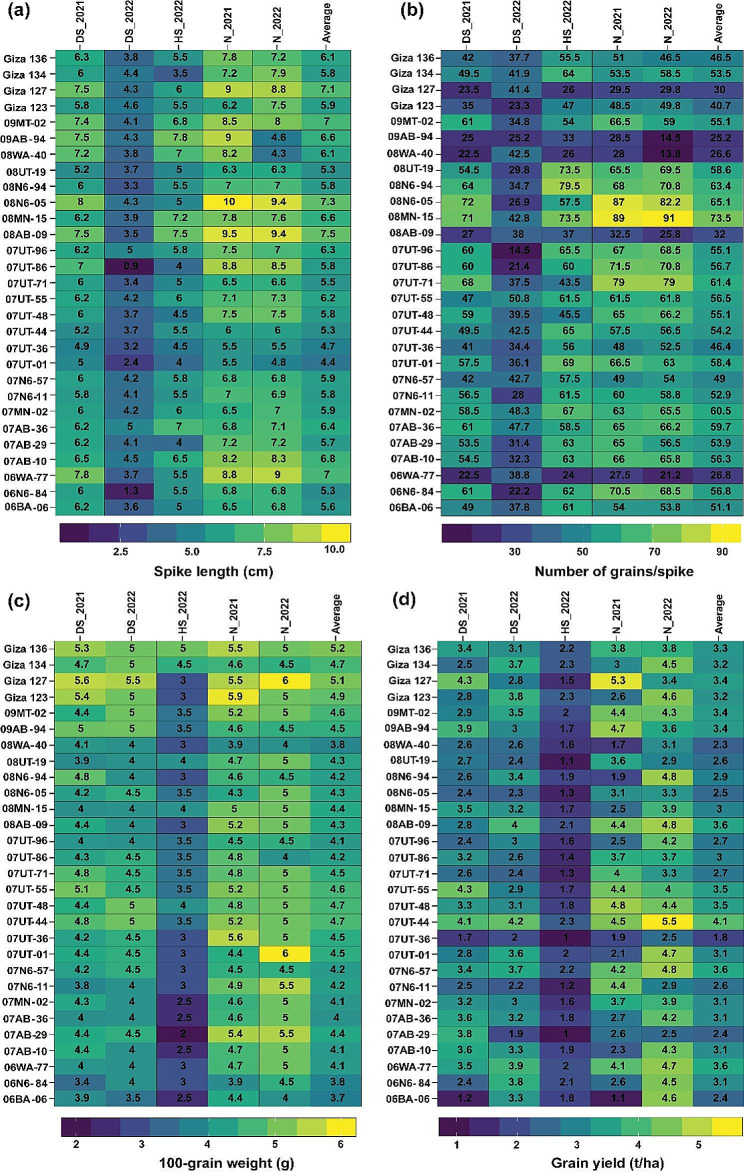
Performance of 29 barley genotypes observed in the five environments for spike length (**a**), number of grains/spike (**b**), 100-grain weight (**c**), and grain yield (**d**). The data represented in each column indicates the average values of each trait of barely genotypes in each of the five environments: normal conditions in 2021 (N_2021) and 2022 (N_2022), drought stress conditions in 2021 (DS_2021) and 2022 (DS_2022) and heat stress conditions (HS_2022).

The performance of the 29 genetic barley genotypes in terms of each of the nine traits in each of the five distinct environments: normal conditions in 2021 (N_2021) and 2022 (N_2022), drought conditions in 2021 (DS_2021) and 2022 (DS_2022), and heat conditions in 2022 (HS_2022) is illustrated in Figs. 2, 3 and 4. The results indicated that contrasting effects were observed on the studied traits, including agronomic, yield, physiological, and pathological traits, when barley genotypes were grown in those diverse environments. It can be figured out that heat- and drought-stressed barley genotypes often show different performances in most traits in comparison to those exhibited under optimal conditions. Also, the response of barley genotypes to heat stress and drought stress varied depending on the genotype, severity, and duration of the stress. The result of the heatmap indicated that PH (Fig. 2a) and NDF (Fig. 2b) demonstrated distinct variations among the five environments.

The 29 genotypes demonstrated comparatively lower plant height performance in heat-stressed environments compared to other environments, averaging 68.7 cm. Conversely, the overall performance of these 29 genotypes was relatively higher under normal conditions, with an average plant height of 141.2 cm. For NDF, the 29 genotypes displayed a reduced time to flowering in the heat-stressed environment (50.2 days) compared to their performance in other environments. Conversely, under normal conditions in 2022, NDF increased, resulting in an average of 101.3 days.

In terms of TCC (Fig. 3a), the barely genotypes performed better, showing higher values under normal conditions in 2022 (with a value of 50.6) compared to their performance under heat stress in the same year (with a value of 33.13). Also, the response of the barely genotypes to different environments varied in terms of CT (Fig. 3b). In 2021, under normal conditions, CT reached its highest value (26.5 °C), while under drought stress in 2022, the average was the lowest recorded, at 14.76 °C. In relation to the susceptibility of various barley genotypes to LR (Fig. 3c), the disease severity exhibited the highest value of 6.83 under normal environmental conditions in 2021. Conversely, the lowest disease score of 2.76 was recorded when the barley genotypes were exposed to drought stress in 2022.

Barley genotypes exhibited varied performance in terms of yield-related traits when subjected to different environmental conditions (Fig. 4a-d). For instance, under normal conditions in 2021 and 2022, SL of the barley genotypes was found to be longer, measuring 7.42 cm and 7.14 cm respectively. However, in the heat-stressed environment, the shortest SL of 3.76 cm was observed. For NGS, the lowest average was exhibited under 35.33 grains/spike, while 58.05 under normal conditions in 2021. In respect to HGW, the lowest average value (3.2 g) was recorded under heat-stressed environment, while the highest average value was 4.83 g under normal conditions in 2021. Variation in yield-related traits is a key reason for the change in grain yield across different environments. Thus, grain yield was found to be maximal under normal conditions (3.98 t/ha), while it was 1.73 t/ha under heat-stressed environment.

### Relationships among barely genotypes

**Fig. 5:**
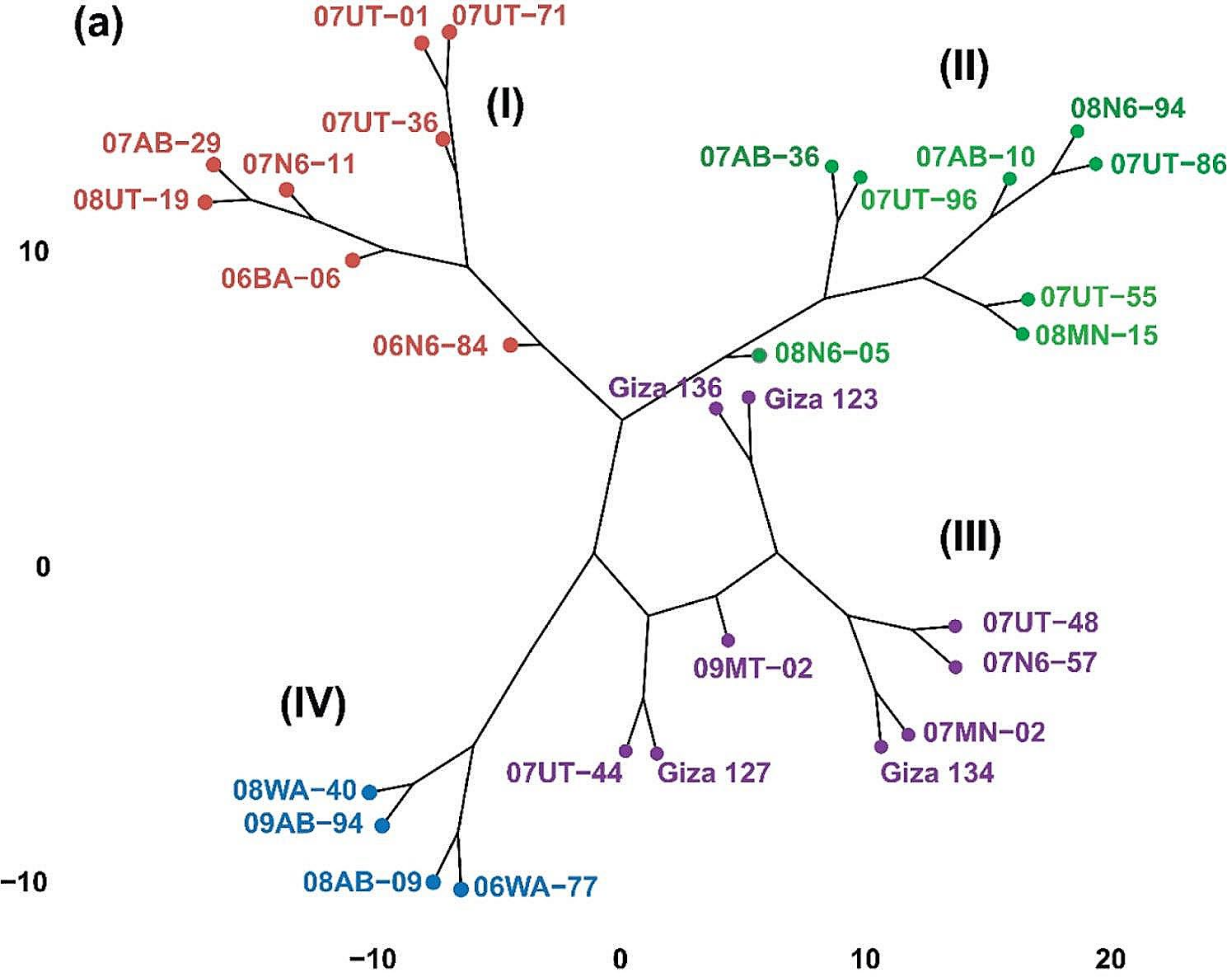
Dendrogram showing hierarchical classification of 29 barley genotypes based on Ward’s method utilizing nine phenotypic traits evaluated across five environments

The interrelationships among 29 barley genotypes were investigated through a cluster analysis, employing Ward’s method, based on their agronomic, physiological, and pathological attributes. The resulting cluster diagram (Fig. 5) demonstrates the grouping of genotypes across five different environments. The analysis revealed four distinct clusters. The first cluster, which constituted eight genotypes (07UT-01, 07UT-36, 07UT-71, 07N6-11, 07AB-29, 06BA-06, 08UT-19, and 06N6-84), represented over 34% of six-row barely genotypes. The second cluster included eight genotypes (07AB-10, 07AB-36, 07UT-55, 07UT-86, 07UT-96, 08MN-15, 08N6-05, and 08N6-94), accounting for 27% of the total genotypes and 34% of all six-row barely type. In the third cluster, nine two-row genotypes (09MT-02 and Giza127) and six-row genotypes (07MN-02, 07N6-57, 07UT-44, 07UT-48, Giza123, Giza134, and Giza136) were clustered together, representing 31% of the total genotypes. Finally, the fourth cluster comprised only two-row barely genotypes, including 06WA-77, 08AB-09, 08WA-40, and 09AB-94, which constituted 13% of the total genotypes.

### Correlation coefficients of studied traits across different environments

**Fig. 6:**
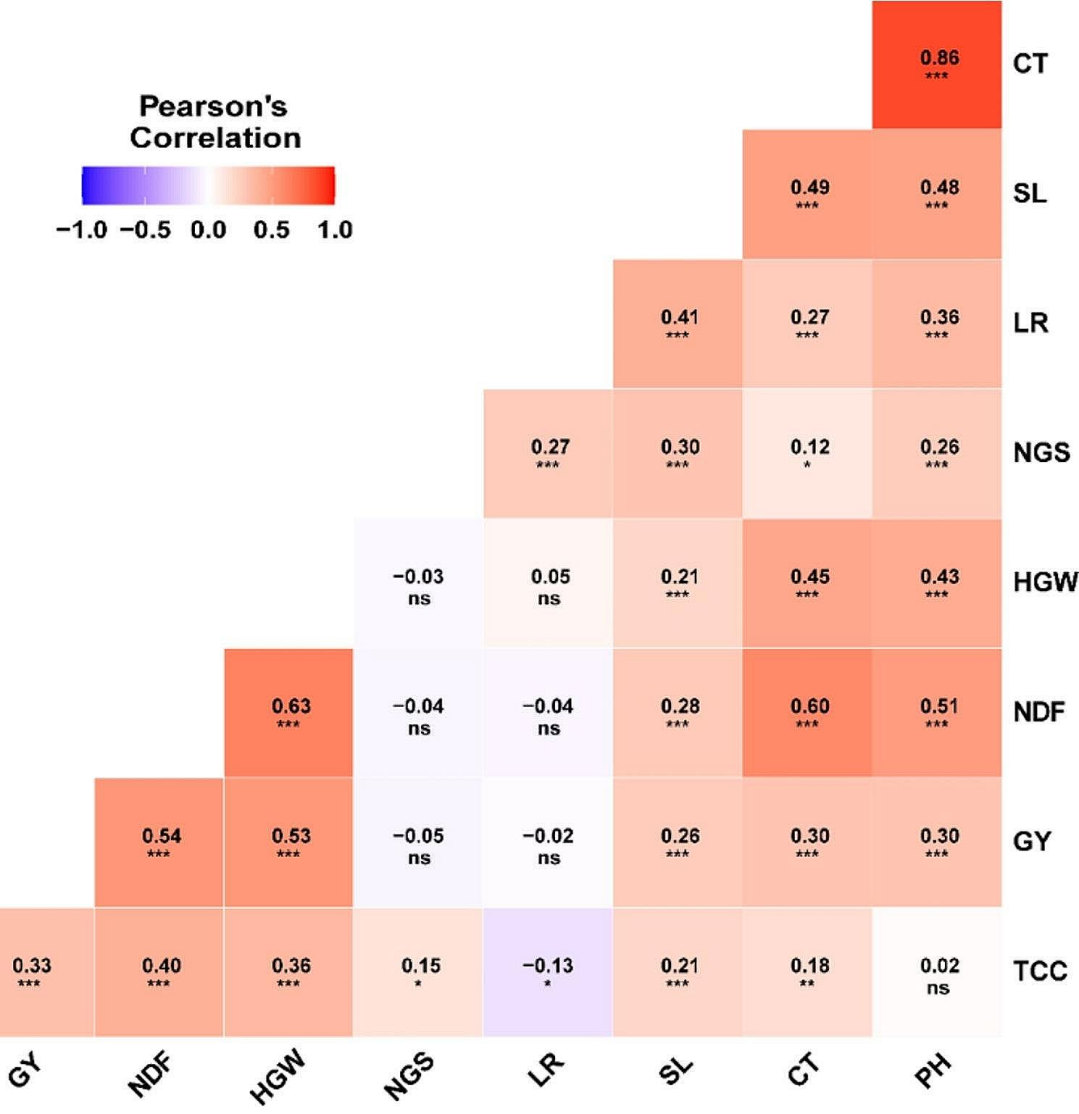
Correlation coefficients between nine agronomic, physiological, and pathological attributes examined in five different environments. NDF, number of days to flowering; PH, plant height; NGS, number of grains/spike; HGW, 100-grain weight; LR, leaf rust; GY, grain yield; CT, Canopy temperatures; TCC, total chlorophyll content; LR, leaf rust. *, **, and ***: Significant at p-value = 0.05, 0.01 and 0.001. ns: means non-significant coefficient (p-value > 0.05)

The correlation coefficients for all the traits across the five different environments are displayed in Fig. 6. Most of the correlation coefficients were significantly positive. The highest correlation coefficients were exhibited by canopy temperature and plant height (r = 0.86***), followed by those between number of days to flowering and 100-grain weight (r = 0.63***), number of days to flowering and canopy temperature (r = 0.60***). Other significant and positive correlations were also observed among number of days to flowering and grain yield (r = 0.54***), 100-grain weight and grain yield (r = 0.53***), and number of days to flowering and plant height (r = 0.51***). A significant and negative correlation coefficient was observed only between leaf rust and total chlorophyll content (r = -0.13*).

### Mean performance vs. stability for barley genotypes

**Fig. 7:**
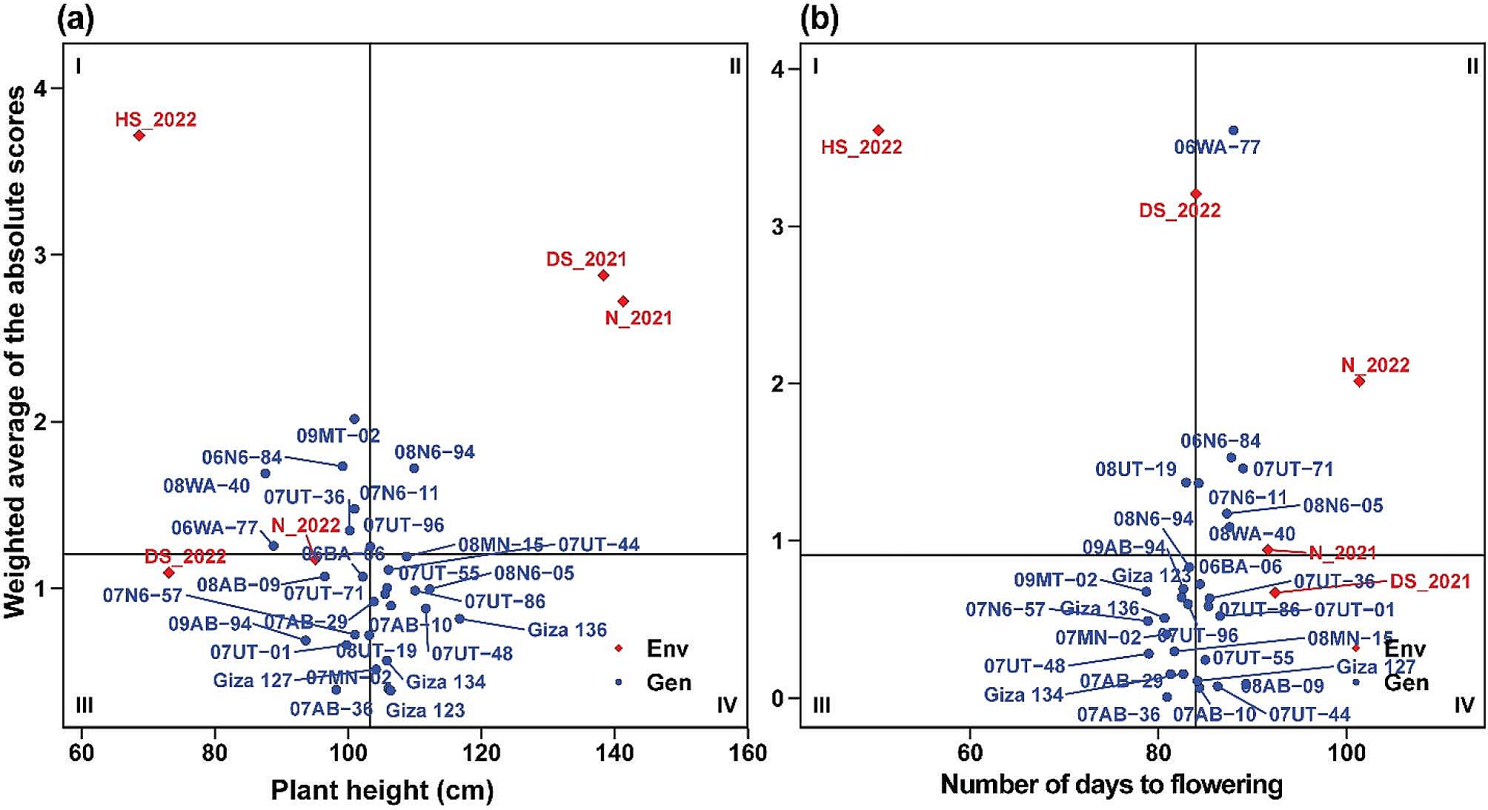
The mean performance vs. WAASB bi-plot shows the joint interpretation of the mean performance of plant height (**a**) and number of days to flowering (**b**) of 29 barely genotypes across five environments

The mean performance vs. weighted average of absolute scores (WAASB) biplot of the 29 barley genotypes for nine agronomic, physiological, and pathological traits is depicted in Fig. 7a-b. This biplot indicates four different cases of performance and stability as classified into quadrants I, 2, 3, and 4. Genotypes positioned in quadrant I demonstrate instability and underperform below the overall average. In quadrant II, genotypes exhibit productivity above the overall average but still lack stability. Quadrant III encompasses genotypes with low productivity, yet they maintain stability, primarily attributed to lower WAASB values. Genotypes in quadrant IV are highly productive and possess broad adaptability.

The results from the mean performance vs. WAASB biplot analysis revealed that among the different genotypes, Giza 123, 07AB-36, and 07MN-02 demonstrated the least variation and high stability in plant height across diverse environments. This was evident from their low WAASB index values of 0.386, 0.391, and 0.398, respectively. Likewise, for the number of days to flowering, it can be noted that 07AB-36, 07AB-10, and 07UT-44 exhibited greater stability, as indicated by their WAASB index values of 0.00675, 0.0641, and 0.0748, respectively. These barley genotypes showed minimal variation and stable performance across those stressed environments in terms of such traits.

**Fig. 8:**
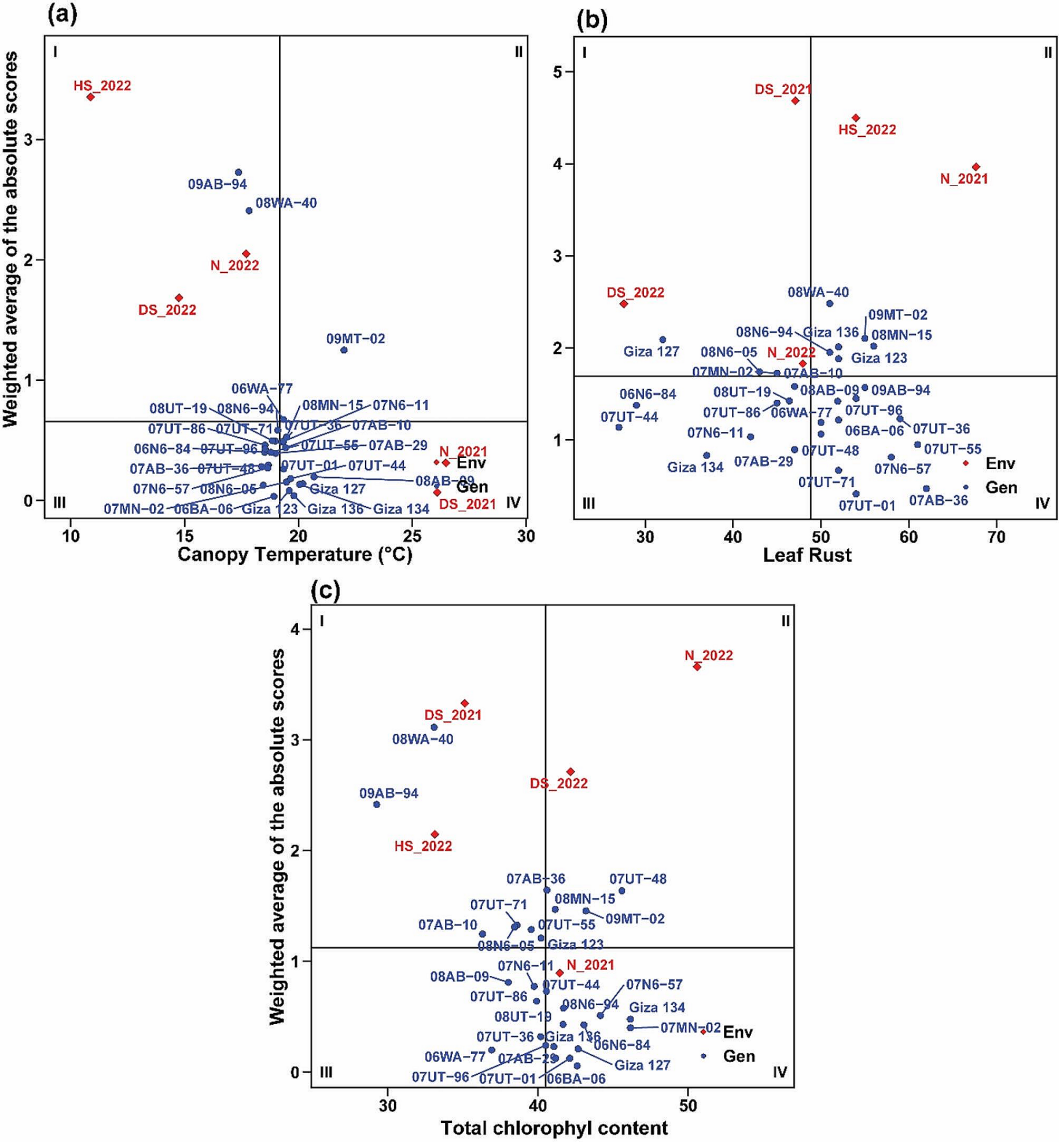
The mean performance vs. WAASB bi-plot shows the joint interpretation of the mean performance of canopy temperature (**a**), leaf rust (**b**), and total chlorophyll content (**c**) of 29 barely genotypes across five environments

The findings regarding physiological and pathological traits demonstrated the notable stability of certain genotypes (Fig. 8a-c). Specifically, the genotypes 06BA-06, Giza 136, and Giza 123 exhibited consistent performance in terms of canopy temperature, as reflected by their WAASB values of 0.0347, 0.0409, and 0.0839, respectively. In terms of total chlorophyll content, the genotypes 06BA-06, 07UT-01, and 07AB-29 displayed the lowest WAASB values of 0.0577, 0.125, and 0.126, respectively, indicating their stable performance. Furthermore, when considering the genotypes’ response to leaf rust susceptibility, the lowest WAASB values were observed in the genotypes 07UT-01 (0.415), 07AB-36 (0.473), and 07UT-71 (0.67), indicating their consistent and minimal variation in coping with the disease under stressed environments. These findings underscore the stability and reliability of these genotypes concerning these specific traits.

**Fig. 9:**
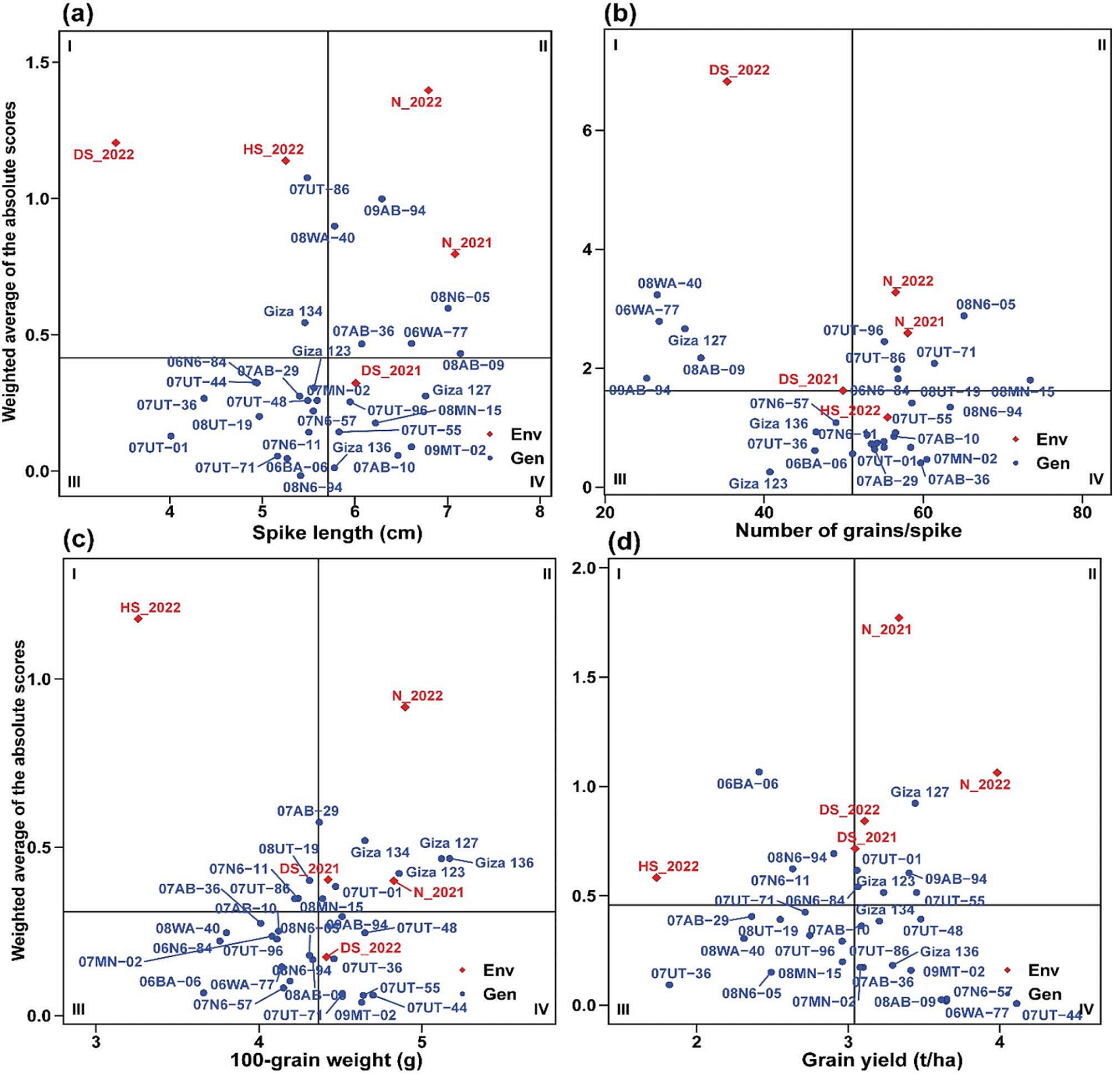
The mean performance vs. WAASB bi-plot shows the joint interpretation of the mean performance of spike length (**a**), number of grains/spike (**b**), 100-grain weight (**c**), and grain yield (**d**) of 29 barely genotypes across five environments

Regarding yield and its attributes, various genotypes exhibited high stability (Fig. 9a-d). In terms of spike length, the genotypes 08N6-94, Giza 136, and 06BA-06 stood out as highly stable, as indicated by their respective WAASB values of 0.0138, 0.0422, and 0.0764 (Figure). In terms of the number of grains per spike, genotypes Giza 123, 07AB-36, and 07MN-02 exhibited the lowest WAASB index values of 0.255, 0.408, and 0.464, respectively. These results indicate a high level of phenotypic stability in this yield attribute. Notably, the trait 100-grain weight demonstrated varying degrees of stability among different genotypes. Notably, genotypes 09MT-02, 07UT-55, and 07UT-44 exhibited the lowest WAASB values of 0.0396, 0.0601, and 0.0608, respectively. These findings indicate a reduced level of variability in this particular trait across different environments. For grain yield, 07UT-44, 06WA-77, 08AB-09, and 07N6-57 demonstrated a high level of stability, as indicated by their low WAASB index of 0.00725, 0.0183, 0.0246, and 0.0277, respectively. Additionally, the genotypes 07UT-44 (4.1 t/ha), 06WA-77 (3.6 t/ha), 08AB-09 (3.6 t/ha), and 07N6-57 (3.6 t/ha) exhibited noteworthy grain yields, surpassing the average yield of all genotypes.

### Selection of genotypes based on multi-trait stability index (MTSI)

**Fig. 10:**
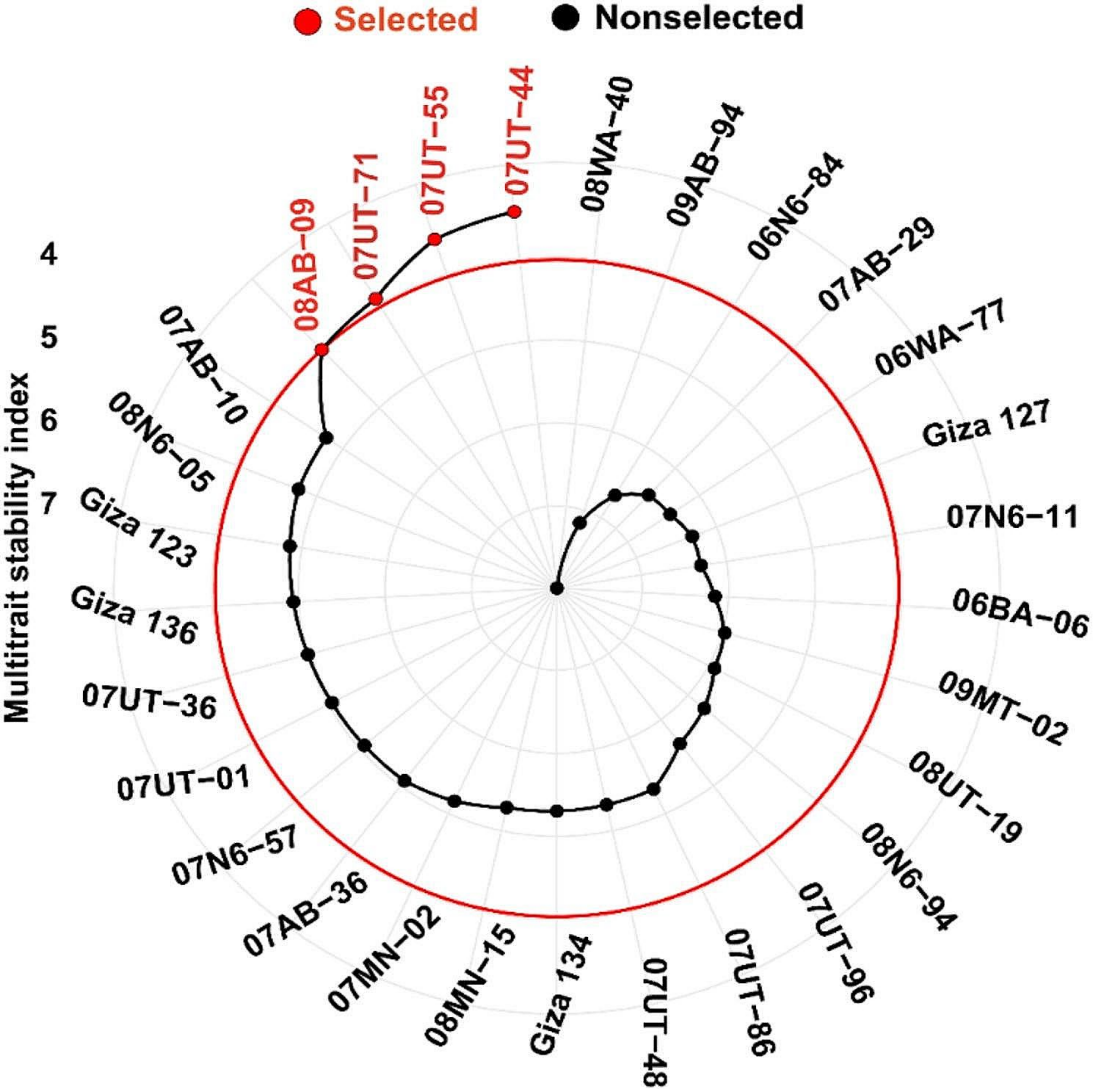
The ranking and selection of the 29 barley genotypes based on the multi-trait stability index, considering nine agronomic traits and five environments, with a selection intensity of 15%

The multi-trait stability index (MTSI) was calculated for 29 barley genotypes based on data from nine traits measured across five different environments (Fig. 10 and Table S3). The MTSI provides a comprehensive assessment of genotype performance by incorporating multiple traits and their stability across diverse environmental conditions. MTSI utilizes the balance between average performance and stability to effectively choose genotypes that exhibit both exceptional performance and stability. The genotype with the lowest MTSI value, indicating the highest stability across multiple traits and environments, was 07UT-44, with an MTSI value of 3.43. This genotype consistently performed well across different environmental conditions, making it a promising candidate for cultivation in various regions. Following 07UT-44, the genotypes 07UT-55 (MTSI = 3.56) and 07UT-71 (MTSI = 3.93) also exhibited low MTSI values, indicating high stability across multiple traits and environments. On the other hand, the genotypes with relatively higher MTSI values, suggesting lower stability, included 08WA-40 (MTSI = 7.99), 09AB-94 (MTSI = 7.16), and 06N6-84 (MTSI = 6.68). These genotypes displayed greater sensitivity to environmental changes and may require further attention and breeding efforts to enhance their adaptability.

## Discussion

To study how different genotypes of barley perform under normal, drought, and heat-stressed conditions, 29 barely genotypes were evaluated, assessing their agronomical, physiological, and leaf rust susceptibility. The results of ANOVA in this investigation indicated that both genotype and environment had a highly significant effect on all the traits examined. These findings were in agreement with those previously reported [27]. Such findings reveal that different genetic makeup of these barley genotypes can have varying inherent characteristics that affect their performance.

The significant effect of the environmental factor was attributed to deleterious impact on barely genotypes, leading to changes in growth, physiology, and disease susceptibility. The interaction between genotype and environment also had a significant impact on most of the traits, except for total chlorophyll content and canopy temperature. This indicates that the performance of genotypes varied across different conditions, as different barley genotypes responded differently to various environmental conditions [28]. Barley genotypes subjected to heat stress and drought stress exhibited distinct performances when compared to their performance under optimal conditions. Noteworthy, these findings are in line with the concept that stress conditions like heat and drought can significantly alter plant physiology and growth patterns [29, 30].

Evidently, both plant height and the number of days to flowering were particularly responsive to the variations in environmental conditions, possibly revealing their role as indicators of stress response or adaptation. Specifically, heat stress conditions have reduced the plant height of the barley genotypes by minimizing vertical growth. This reduction in plant height could be attributed to various physiological mechanisms, such as compromised cell elongation and division under high-temperature conditions [31]. This indicates that, under favorable temperature conditions, these barley genotypes can reach their full growth potential in terms of height, underscoring the significance of temperature as a determinant of plant growth. Similarly, in heat-stressed environments, the barley genotypes demonstrated an accelerated flowering process, with an average NDF of 50.2 days. This suggests that the plants tend to flower earlier when exposed to heat stress, which might be a survival mechanism to complete their life cycle before the stressful conditions worsen [32]. Conversely, under normal conditions, the time required for flowering increased significantly. In the 2022 season, the average NDF was reported to be 101.3 days. This delayed flowering under normal conditions might be associated with the plants having more favorable conditions for growth and development, allowing them to allocate more resources towards vegetative growth before initiating the reproductive phase.

The results in this study also revealed that heat stress had a negative impact on the chlorophyll content of the barley plants, which aligns with what was documented in recent studies on barley [33, 34]. Chlorophyll is essential for photosynthesis, and a decline in its content can affect the plant’s ability to produce energy from sunlight. Moreover, varying responses in canopy temperature among barley genotypes to various stress conditions were also observed in the current investigation. Lower canopy temperatures under drought stress could indicate reduced water loss through transpiration, which is a survival strategy for plants to conserve water during periods of limited water availability [35]. Basically, leaf rust is a common disease affecting barley plants [36], so the susceptibility of different barley genotypes to this disease under different environmental conditions was assessed in this investigation. The study found that disease severity was highest under normal environmental conditions in 2021, with a score of 6.83. On the other hand, the lowest disease score of 2.76 was recorded when the barley genotypes were exposed to drought stress in 2022. This indicates that drought stress might have a positive effect on reducing the severity of leaf rust in barley plants. This finding could have important implications for disease management strategies in regions where both drought stress and leaf rust are significant concerns.

The correlation analysis was performed in this study to examine the relationship among the various studied traits across different environments. The highest correlation coefficient was reported between canopy temperature and plant height, indicating a strong positive relationship between these two traits. This suggests that there might be a physiological or genetic connection between these traits. The positive correlation recorded between the number of days to flowering and canopy temperature reveals the potential influence of temperature on the flowering process, as plants that flower later often invest more time and resources in their growth and development prior to flowering. This prolonged growth duration can result in increased grain yields, including greater grain weight [37–39]. The significant and negative correlation coefficient reported between leaf rust and chlorophyl further suggests the inverse relationship between these two variables, where increasing the severity of leaf rust led to a reduction in chlorophyll levels. The susceptibility of barley plants to leaf rust can result in various physiological changes within the plant, including a reduction in chlorophyll production. Moreover, the increasing susceptibility to leaf rust might allocate resources differently, which could lead to a decrease in chlorophyll production. Previous reports were in line with those observed in our current study [40]. According to the findings of [41, 42], the infection of winter wheat with yellow rust led to a reduction in the content of chlorophyll in its leaves.

Cluster analysis provides a comprehensive overview of the relationships among the barley genotypes based on their agronomic, physiological, and pathological attributes. In this study, the cluster analysis conducted on the 29 barley genotypes provided valuable insights into their relationships based on agronomic, physiological, and pathological attributes. The identification of distinct clusters highlights the diversity and variability present within the set of genotypes studied. Genotypes within each of the four clusters suggest a high degree of similarity among these genotypes in terms of their agronomic, physiological, and pathological characteristics. The third cluster displayed a mix of nine two-row and six-row barley genotypes, encompassing both genetic types and suggesting that, despite genetic differences, these genotypes share similar agronomic, physiological, and pathological characteristics. The findings of cluster analysis in this study can be valuable for further exploration and utilization of the genotypes in breeding programs [43].

The mean performance vs. WAASB biplot for the 29 barley genotypes was assessed for various variables across five environments. Several barley genotypes exhibited distinct characteristics, with low WAASB values and high performance. Notably, 07UT-44, 06WA-77, 08AB-09, and 07N6-57 were observed as promising genotypes due to their high grain yield coupled with high stability. These findings underscore the phenotypic stability and performance of specific barley genotypes across varying stress conditions and environments. Several recent studies have been carried out on barley, utilizing the WAASB index, which has demonstrated its effectiveness in identifying barley genotypes that consistently exhibit high yields across diverse environmental conditions [44, 45]. The identification of genotypes with high stability is indeed crucial for sustainable barley cultivation. This is important because barley is grown in a range of agro-climatic conditions, each presenting unique environmental challenges [20, 46].

According to the findings in this work, genotypes with lower MTSI values demonstrated consistent performance across multiple traits and environments, making them desirable for breeding programs. For instance, genotype 07UT-44 showed remarkable stability, indicating its adaptability to diverse environments. Further investigations into the genetic basis underlying its stability could offer valuable insights for developing stable barley varieties. On the other hand, genotypes with higher MTSI values exhibited lower stability, suggesting the need for improvement in their adaptability. For instance, genotypes 08WA-40, 09AB-94, and 06N6-84 displayed higher sensitivity to environmental changes, indicating a lower degree of stability. Breeding efforts focusing on enhancing their performance and adaptability under diverse conditions may be warranted. The power of MTSI to discern robust soybean genotypes displaying stability under both drought and salinity stress scenarios was also recently harnessed [18]. Moreover, MTSI was effectively employed to pinpoint five sugar beet genotypes that exhibited resilience in the face of rhizomania disease within field conditions [19]. These results agree with the current investigation’s own findings, underscoring the efficacy of MTSI in the identification of elite genotypes.

## Conclusion

This study provides valuable insights into how stressed environments affect barley genotypes and highlights the importance of understanding such impacts for maintaining consistent productivity in the face of climate change-induced stresses. The evaluation of 29 barley genotypes under various normal and stressed conditions (drought and heat) shed light on the complex genotype-by-environment interactions influencing agronomic, physiological, and pathological traits. As evident from their mean performance vs. stability index (WAASB), the four barley genotypes: 07UT-44, 06WA-77, 08AB-09, and 07N6-57 showed lower WAASB values in terms of grain yield, thereby exhibiting not only the highest grain yield but also greater stability across different environments. Furthermore, the multi-trait stability index identified genotypes 07UT-44, 07UT-55, 07UT-71, and 08AB-09 as the most stable for all evaluated traits and across all environments. It’s worth noting that the distinct imported barley lines consistently exhibited better performance and high stability in this research. This suggests that these barley resources can be utilized as parent materials to improve barley production and enhance genetic diversity. Additionally, after undergoing rigorous testing, they might also be candidates for direct introduction as new barley cultivars in Egypt.

While these findings showcase promising heat- and drought-tolerant barley genotypes, further multi-location, multi-year evaluations are imperative to validate stability. Our future efforts will concentrate on crossing the best-performing exotic lines with adapted local cultivars to combine stress resilience with yield potential. Advanced breeding populations from such strategic crosses will undergo simultaneous selection for productivity, quality, and enhanced climate resilience to accelerate the development of farmer-preferred barley varieties tailored for Egypt.

### Electronic supplementary material

Below is the link to the electronic supplementary material.

Supplementary Material 1

## Data Availability

All the data are available in the manuscript and with Correspondence authors.

## Abbreviations

MTSI: Multi-trait stability index
WAASB: Weighted Average of Absolute Scores
NDF: Number of days to flowering
PH: Plant height
SL: Spike length
NGS: Number of grain per spike
HGW: Seed weight
GY: Grain yield
TCC: Total chlorophyl content
CT: Canopy temperature
LR: Leaf rust
ANOVA: Analysis of variance
GEN: Genotype
ENV: Environment
CV: Coefficient of variation
DS: Drought stress
HS: Heat stress
