# Supplementary material for: Dissecting the resilience of barley genotypes under multiple adverse environmental conditions
Source: BMC Plant Biol. 2024 Jan 2;24:16. doi: 10.1186/s12870-023-04704-y (PMC10759481; doi:10.1186/s12870-023-04704-y)
Supplement: Supplementary file 1 — Supplementary Material 1 [file 12870_2023_4704_MOESM1_ESM.docx]

**Table S1.** Physical and chemical soil properties for El- Bostan in 2020/2021 and 2021/2022 growing seasons.

| **Environments Physical properties** | **2020/2021** | **2021/2022** |
| --- | --- | --- |
| Clay% | 0.9 | 1.1 |
| Silt% | 1.5 | 1.4 |
| Sand% | 97.6 | 97.5 |
| Soil texture | Sand | Sand |
| Chemical properties | value | value |
| pH | 7.7 | 8.5 |
| EC(dsm^-1^) | 1.85 | 0.71 |
| CaCO3 | 6.1 | 4.16 |
| Organic matter % | 0.04 | 0.05 |
| Soluble cations meq100^-1^ g soil | | |
| Ca++ | 6.1 | 1.64 |
| Mg++ | 3.0 | 1.67 |
| Na++ | 9.5 | 3.49 |
| K+ | 0.1 | 1.87 |
| Soluble anions meq100^-1^ g soil | | |
| HCO3 | 1.8 | 2.1 |
| Cl^-^ | 9.8 | 3.6 |
| SO4 | 7.1 | 1.48 |

1

**Table S2. Meteorological data^*^ for El-Bostan experimental farm in 2020/2021 and 2021/2022 growing seasons.**

|  | **Ave. Temperature ◦C** | | **Max. Temperature ◦C** | | **Min. Temperature ◦C** | | **Precipitation (mm)** | | **Relative humidity (%)** | | **Wind speed (km/h)** | |
| --- | --- | --- | --- | --- | --- | --- | --- | --- | --- | --- | --- | --- |
|  | 2020/21 | 2021/22 | 2020/21 | 2021/22 | 2020/21 | 2021/22 | 2020/21 | 2021/22 | 2020/21 | 2021/22 | 2020/21 | 2021/22 |
| OCT | 25.8 | 24.38 | 33 | 35.53 | 16.97 | 16.23 | 0 | 1.91 | 58.62 | 56.88 | 2.51 | 2.7 |
| NOV | 19.19 | 21.11 | 28.53 | 33.18 | 10.49 | 13.01 | 5.27 | 36.8 | 64.5 | 63.88 | 2.37 | 2.23 |
| DEC | 16.17 | 14.26 | 26.23 | 24.18 | 8.58 | 6.8 | 0 | 22.45 | 63.94 | 69.25 | 2.18 | 2.67 |
| JAN | 14.62 | 12.87 | 27.06 | 23.8128 | 5.2 | 4.58 | 0 | 33 | 63.88 | 67.7128 | 2.58 | 2.7348 |
| FEB | 14.6 | 12.85 | 27.96 | 24.6048 | 6.12 | 5.39 | 31.46 | 52 | 65.06 | 68.9636 | 2.25 | 2.385 |
| MAR | 15.89 | 13.98 | 33 | 29.04 | 5.87 | 5.17 | 68.55 | 22 | 63.19 | 66.9814 | 2.74 | 2.9044 |
| APR | 19.94 | 17.55 | 40.89 | 35.9832 | 7.4 | 6.51 | 0.44 | 0 | 53.88 | 57.1128 | 2.98 | 3.1588 |
| MAY | 26.63 | 23.43 | 42.72 | 37.5936 | 15.07 | 13.26 | 0 | 0 | 44.12 | 46.7672 | 2.91 | 3.0846 |

* Source: Central Laboratory for Agricultural Climate (CLAC), Cairo, Egypt.

**Table S3. Multiple trait stability index (MTSI) for 29 barley genotypes across five environments.**

| **Genotype** | **MTSI** | **Genotype** | **MTSI** |
| --- | --- | --- | --- |
| 07UT-44 | 3.43 | 07UT-48 | 5.32 |
| 07UT-55 | 3.56 | 07UT-86 | 5.32 |
| 07UT-71 | 3.93 | 07UT-96 | 5.64 |
| 08AB-09 | 4.04 | 08N6-94 | 5.75 |
| 07AB-10 | 4.77 | 08UT-19 | 5.92 |
| 08N6-05 | 4.77 | 09MT-02 | 5.97 |
| Giza 123 | 4.86 | 06BA-06 | 6.16 |
| Giza 136 | 4.94 | 07N6-11 | 6.3 |
| 07UT-36 | 5 | Giza 127 | 6.3 |
| 07UT-01 | 5.05 | 06WA-77 | 6.41 |
| 07N6-57 | 5.07 | 07AB-29 | 6.44 |
| 07AB-36 | 5.08 | 06N6-84 | 6.68 |
| 07MN-02 | 5.17 | 09AB-94 | 7.16 |
| 08MN-15 | 5.29 | 08WA-40 | 7.99 |
| Giza 134 | 5.31 |  |  |
